# Exploration and confirmation of the latent variable structure of the Jefferson scale of empathy

**DOI:** 10.5116/ijme.533f.0c41

**Published:** 2014-04-20

**Authors:** Mohammadreza Hojat, Marianna LaNoue

**Affiliations:** 1 Jefferson Medical College of Thomas Jefferson University, Center for Research in Medical Education and Health Care, and Department of Psychiatry and Human Behavior, USA; 2 Jefferson Medical College of Thomas Jefferson University, Department of Family and Community Medicine, USA

**Keywords:** Jefferson Scale of Empathy, exploratory factor analysis (EFA), confirmatory factor analysis (CFA), latent variable structure

## Abstract

Objectives: To reaffirm the underlying components of the JSE by using exploratory factor analysis (EFA), and to confirm its latent variable structure by using confirmatory factor analysis (CFA).

**Methods:**

Research participants included 2,612 medical students who entered Jefferson Medical College between 2002 and 2012. This sample was divided into two groups: Matriculants between 2002 and 2007 (n=1,380) and between 2008 and 2012 (n=1,232). Data for 2002-2007 matriculants were subjected to EFA (principal component factor extraction), and data for matriculants of 2008-2012 were used for CFA (structural equation modeling, and root mean square error for approximation).

**Results:**

The EFA resulted in three factors: “perspective-taking,” “compassionate care” and “walking in patient’s shoes” replicating the 3-factor model reported in most of the previous studies. The CFA showed that the 3-factor model was an acceptable fit, thus confirming the latent variable structure emerged in the EFA. Corrected item-total score correlations for the total sample were all positive and statistically significant, ranging from 0.13 to 0.61 with a median of 0.44 (p<0.01). The item discrimination effect size indices (contrasting item mean scores for the top-third versus bottom-third JSE scorers) ranged from 0.50 to 1.4 indicating that the differences in item mean scores between top and bottom scorers on the JSE were of practical importance. Cronbach’s alpha coefficient of the JSE for the total sample was 0.80, ranging from 0.75 to 0.84 for matriculatnts of different years.

**Conclusions:**

Findings provided further support for underlying constructs of the JSE, adding to its credibility.

## Introduction

Empathy is an essential element of clinical competence and professionalism in medicine.^1^ Based on an extensive review of the literature on personality assessments and outcomes in medical education, empathy was identified as one of the most pertinent aspect of personality in health profession education and patient care.^2^ Because of the importance of empathy in the development of physicians-in-training, the Medical School Objectives Project of the Association of American Medical Colleges^3^ recommended that enrichment of interpersonal skills and empathy be included among the educational objectives of undergraduate medical education. Also, in a position paper, the American Board of Internal Medicine^4^ recommended that humanistic values and empathy should be assessed and cultivated as an essential educational activity in graduate medical education. These recommendations are supported by empirical research. For example, research has shown that medical students with high empathy scores obtained better ratings of global clinical competence given by medical school faculty in core clerkships.^5^ Also, physicians with high empathy scores had more optimal clinical outcomes in their diabetic patients.^6^^,^^7^ It has been suggested that a combination of psych-socio-bio-neurological mechanisms could explain the observed link between physician empathy and patient outcomes.^8^

### Conceptualization of empathy

Empathy is an ambiguous concept. Despite a lack of consensus about its definition, there are various descriptions or characterizations of the term in the literature.^9^ Because of this conceptual ambiguity, empathy has been described as a notion that is difficult to define and hard to measure.^10^ Generally, some researchers have described empathy as a cognitive attribute,^11^^,^^12^ which implies that it predominantly involves understanding another person’s concerns. Others have described empathy as an affective or emotional characteristic,^13^^,^^14^ which implies that it primarily involves feeling another person’s pain and suffering. Yet, there is a third group that views empathy as both affective and cognitive.^15^^,^^16^To clarify the conceptual ambiguity associated with empathy, based on an extensive review of relevant literature, empathy in the context of medical education and patient care was defined as a predominantly cognitive (as opposed to affective or emotional) attribute that involves an understanding (as opposed to feeling) of patients’ experiences, concerns, and perspectives combined with a capacity to communicate this understanding and an intention to help by preventing and alleviating pain and suffering.^9^^,^^17^^,^^18^ The key terms in this definition are italicized for two reasons: (1) to underscore their importance in the construct of empathy in the context of health profession education and patient care, and (2) to make a distinction between empathy and sympathy, which have often been mistakenly used interchangeably.^9^

### Empathy versus sympathy

Sympathy, in contrast to cognitively defined empathy, is predominantly an affective or emotional (as opposed to cognitive) attribute that involves intense feelings (as opposed to understanding) of a patient’s pain and suffering. Despite the differences in conceptualization, the two notions are not entirely independent and can be measured.^19^ Based on the aforementioned conceptualization, sympathy is analogous to what others described as emotional empathy, affective empathy, and vicarious emotional empathy.^19^Although the interchangeable use of these two concepts may not cause a problem in social psychology, it is important to separate the two in the context of patient care. In social psychology, both empathy and sympathy can lead to a similar outcome (e.g., prosocial behavior), albeit for different behavioral motivations. For example, a prosocial behavior that is induced by empathic understanding is more likely to be elicited by a sense of altruism. A prosocial behavior that is prompted by sympathetic feelings is more likely to be triggered by egoistic motivation to reduce personal distress.^9^In the context of medical education and patient care, however, we must make a distinction between the two concepts because, in this context, they lead to different clinical behavior and patient outcomes.^20^ An empathic physician would be more concerned about understanding of the kind and quality of patients’ experiences, whereas a sympathetic physician would be more concerned about feeling the degree and intensity (quantity) of patients’ pain and suffering.^9^ Because of its cognitive nature, abundance of empathy is always beneficial in patient-physician relationships; understanding in excess cannot be detrimental.In contrast, because of its affective nature, sympathy in excess can be disadvantageous to patient-physician relationships. Emotions in excess can impede the neutrality that is necessary in clinical decision making, thus negatively influencing a physician’s performance.^9^ Cognitively defined empathy can lead to personal growth, career satisfaction, and optimal clinical outcomes,^9^^,^^19^ whereas affectively defined sympathy can lead to career burnout, compassion fatigue, exhaustion, and vicarious traumatization.^21^ Empathy is rooted in reasoning and logic, thus binding the patient and caregiver together based on mutual understanding. In contrast, sympathy can be fed by irrational emotions; thus, empathy binds, sympathy blinds!Indeed, it can be assumed that the relationship between empathy and positive clinical outcomes is linear, meaning that the outcomes progressively become better as a function of an increase in empathy.^9^^, ^^19^ In contrast, it can be assumed that the relationship between sympathy and clinical outcomes is like an inverted U shape (similar to that between anxiety and performance), meaning that sympathy to a limited extent can be beneficial, but excessive sympathy can be detrimental to the patient-physician relationship and patient outcomes.^9^^, ^^19^Another important implication for making a distinction between empathy and sympathy in medical education is the fact that affect and emotion (the prominent ingredients of sympathy) are less amenable to change, whereas cognition and understanding (the prominent ingredients of empathy) can be substantially enhanced through education.^9^ Specific features of empathy compared to sympathy have been described in more detail elsewhere.^9^^,^^18^

### Measurement of empathy

To the best of our knowledge, prior to the development of the Jefferson Scale of Empathy (JSE), no psychometrically sound instrument was available to measure empathy specifically among medical students, residents, physicians, and other health profession students and practitioners. Although a few research tools exist for measuring empathy,^9^ none is content-specific to medical education and context-relevant to patient care.These tools were developed for administration to the general population. The following Four have often been used in medical education research. 1) The Interpersonal Reactivity Index (IRI) which was developed by Davis^15^ and includes 28 items tapping both cognitive and emotional empathy. The IRI contains four scales: perspective-taking, empathic concern, fantasy, and personal distress. A typical item (from the perspective-taking scale) is: “I sometimes try to understand my friends better by imagining how things look from their perspective.” 2) The Empathy Scale developed by Hogan^22^ which includes 64 items. A typical item is: “I have seen some things so sad that I almost felt like crying.” 3) The Emotional Empathy Scale developed by Mehrabian and Epstein^23^ which includes 33 items intended to measure “emotional empathy.” A typical item is: “It makes me sad to see a lonely stranger in a group.” 4) Mehrabian introduced a new 30-item instrument, the Balanced Emotional Empathy Scale (BEES)^24^ adapted from the Emotional Empathy Scale to measure vicarious emotional empathy. A sample item is: “Unhappy movie endings haunt me for hours afterward.”As indicated before, and reflected in the content of sample items, none of the aforementioned instruments features content specific to medical education and patient care, thus raising concerns about their face and content validities in the context of patient care. With the exception of the BEES, extensive psychometric data from the general population and college students have been published for the other three instruments.^9^ There are other instruments for measuring empathy in children and in the general population, some of which are described elsewhere.^9^

### The Jefferson Scale of Empathy

Several years ago, one of the authors (MH) and his colleagues at Jefferson Medical College recognized a need for an instrument to measure empathy in the context of medical education and patient care. In response to this need, based on a comprehensive review of the literature and with regard to the above-mentioned cognitively-defined empathy, the Jefferson Scale of Empathy (JSE) was developed.^9^^, ^^25^^, ^^17^The original scale was known as the Jefferson Scale of Physician Empathy (JSPE),^9^^,^^25^ but was renamed Jefferson Scale of Empathy for administration to a broader population of all health profession students and practitioners.^9^^,^^17^ Step-by-step procedures in the development of the JSE and data in support of its validity and reliability are reported elsewhere.^9^ The scale is brief and includes 20 items answered on a 7-point Likert-type scale (Strongly Agree=7, Strongly Disagree = 1) which can be completed in less than 10 minutes. To control for the “acquiescence” response style (a tendency to passively and consistently endorse “agree” [or “disagree”] responses to the test questions), 10 items are positively worded (directly scored) and 10 items are negatively worded (reverse scored). The possible range of scores is 20-140, the higher the score the more empathic orientation toward patient care. The JSE has received broad attention and has been translated into 45 languages to date, and used in more than 70 countries.Three versions of the JSE are available: one for administration to medical students (S-Version), another for administration to physicians and other health professionals (HP-Version), and the third for administration to students in any health profession discipline other than medicine (HPS-Version). These versions are similar in content with slight changes in wording to reflect students’ orientation toward empathy in medical education (S-Version), in other health profession education (HPS-Version), and behavioral tendencies toward empathic engagement in patient care (HP-Version). For example, an item in the S-Version that reads, “Patients feel better when their physicians understand their feelings,” reads as, “Patients feel better when their health care providers understand their feelings,” in the HPS-Version, and reads as, “My patients feel better when I understand their feelings” in the HP-Version. Evidence in support of the JSP’s construct validity,^9^^,^^25^^,^^17^ criterion-related validity,^25^^,^^5^ predictive validity,^26^ internal consistency reliability,^25^^,^^17^ and test-retest reliability^17^ has been reported.

### Factor analytic studies of the JSE

Exploratory factor analytic research of the JSE in physicians^17^ resulted in three underlying factors. The prominent factor of the scale involves a construct entitled “perspective-taking,” which is considered an important ingredient of empathy.^9^^,^^17^ The second component of the JSE, “compassionate care” which is defined as a combination of empathy and sufficient degree of sympathy,^9^ is considered an essential dimension of the patient-physician relationship.^9^^, ^^17^ The third component is “walking in patient’s shoes.”Exploratory factor analytic studies of the JSE by researchers in the United States and abroad have often resulted in the three aforementioned factors. For example, Ward and her colleagues^27^ in their study with 333 nursing students reported the three aforementioned factors. Fjortoft and her colleagues^28^ reported the two factors of “perspective-taking” and “compassionate care” in a study with 187 pharmacy students. In a study with 130 dental school students by Sherman and Cramer,^29^ four factors emerged replicating the original 3-factor model plus a residual factor.The three factors of “perspective-taking” “compassionate care” and “walking in patient’s shoes” have also emerged in studies abroad: in Mexico with 1,022 medical students;^30^ in Japan with 400 medical students;^31^ in Korea with 493 medical students;_32_ in South Africa with 164 medical students;^33^ in China with 1,200 physicians^34^, and with 902 Chinese medical students^35^; in Taiwan with 613 Taiwanese nursing students;^36^ in Portugal with 476 Portuguese medical students;^37^ and in Iran with 180 physicians^38^ and 181 medical students.^39^ Paro and her colleagues^40^ in a study with 299 Brazilian medical students discerned the three factors but in a different order (e.g., “standing in patient’s shoes” as the first and “perspective-taking” as the third factor).Tavakol and colleagues^41^ reported a three factor solution using data for 853 British medical students; however, they entitled the third factor as “emotional detachment” (probably because of negative wordings) rather than “walking in patient’s shoes.” Suh and his colleagues^42^ reported only the two factors of “compassionate care” and “perspective-taking” plus a residual factor in a study with 229 Korean physicians. Williams and his colleagues^43^ in Australia found a 2-factor solution (“perspective-taking” and “compassionate care”) in 330 paramedic students, and Preusche and Wagner-Menghin^44^ reported a 4-factor solution which included the three above-mentioned factors plus one residual factor in 516 Austrian medical students (German translation).There are only a few confirmatory factor analytic studies of the JSE. Tavakol and colleagues^40^ tested the 3-factor model in a sample of 853 British medical students and found good model fit. They concluded that the 3-factor model (and non-orthogonal) structure of the scale was supported by the excellent model fit. Shariat & Habibi^45^ used a sample of 1,187 Iranian medical students and found support for the 3-factor non-orthogonal model. However the fit indices were moderately less than those reported in the Tavakol et al^41^ study. Williams, et al^43^ in a sample of 330 Australian paramedical students tested the 2-factor solution which emerged in their exploratory factor analytic study and reported a relatively poor model fit which necessitated the constraining of several model coefficients in order to improve the fit model.

### Purpose of the study

The aforementioned factor analytic studies provide clues about the underlying components of the JSE in various samples of different disciplines and in a variety of cultures. However, despite the accumulating evidence, it is desirable to undertake additional large scale exploratory and confirmatory factor analytic research, using split samples from the same population to reaffirm the underlying components of the JSE, and to further confirm its latent variable structure. This study was designed to serve that purpose.

## Methods

This study was approved by the Institutional Review Board of Thomas Jefferson University, as part of the Jefferson Longitudinal Study of medical Education. This is a correlational ex post facto design study.

### Study participants

Total participants included 2,612 students who entered Jefferson Medical College in the past 11 years (between 2002 and 2012). These students completed the JSE plus a set of other surveys at the beginning of medical school. This sample represents 93% of all first-year matriculants during the study period (N=2,802). There were 1,322 women (51%) and 1,290 (49%) men in this sample.

### Statistical analyses

We used Pearson correlation coefficients to examine relationships between scores of each item and the total score of the JSE. For that purpose, we calculated the corrected item-total score correlations (by excluding the corresponding item from the total JSE score). To address the discrimination power of each item, we calculated an item discrimination effect size index. For that purpose, we divided the total sample into two groups of approximately top-third high scorers on the JSE (score > 119, n=835) and bottom-third low scorers (JSE score < 111 < n=857). For each item, we calculated the mean score difference between the top-third and bottom-third JSE scoring groups, divided by the pooled standard deviation of the item to calculate the item discrimination effect size index, similar to the Cohen’s *d* ^46^ (Item discrimination effect size index=*M_top-third_–M_bottom-third_)/pooled SD*).

We conducted both exploratory and confirmatory factor analysis. For factor analytic studies we divided the sample into two groups: 1) Matriculants between 2002-2007 (n=1,380); data from this group were used for exploratory factor analysis (EFA). 2) Matriculants between 2008-2012 (n=1,232); data from this group were used for confirmatory factor analysis (CFA). We used principal component factor extraction with oblique rotation in our exploratory factor analysis to re-examine the underlying components of the JSE. For confirmatory factor analysis we used structural equation modeling (SEM) and root mean square error for approximation (RMSEA)^47^ to confirm the latent variable structure of the scale.

## Results

### Item statistics

Respondents used the full range of possible answers (1-7) for each item. Item mean scores ranged from a low of 3.6 (SD=1.4) for this item: “Physicians should not allow themselves to be influenced by strong personal bonds between their patients and their family members” to a high of 6.5 (SD=0.8) for this item: “Patients feel better when their physicians understand their feelings.”

### Item-total score correlations

The corrected item-total score correlations ranged from a low of 0.13 (for the aforementioned item with the lowest mean score) to a high of 0.61 (for this item: "Physicians’ understanding of the emotional status of their patients, as well as that of their families is one important component of the physician-patient relationship.” The median item-total score correlation was 0.44. All correlations were positive and statistically significant (p< 0.01) which indicates that all items contribute positively and significantly to the total score of the JSE scale. Item-total score correlations are reported in Table 1.

**Table1:** Rotated factor pattern for the Jefferson scale of empathy*, item-total score correlations, and effect size estimates of item discrimination indices (n=1,380)

Items^†^	Factors			Item-total score correlation^‡**^	Discrimination Index effect size^‡^
	Factor 1	Factor 2	Factor 3		
Patients value a physician’s understanding of their feelings which is therapeutic in its own right.(10)	0.66	0.02	0.01	0.55	1.3
Physicians should try to stand in their patients’ shoes when providing care to them.(9)	0.64	-0.05	0.02	0.50	1.2
Physicians should try to think like their patients in order to render better care.(17)	0.61	-0.16	0.00	0.37	1.0
Physicians’ understanding of the emotional status of their patients, as well as that of their families is one important component of the physician-patient relationship.(16)	0.46	0.29	0.00	0.61	1.4
I believe that empathy is an important therapeutic factor in medical treatment.(20)	0.44	0.26	-0.02	0.59	1.3
Patients feel better when their physicians understand their feelings.(2)	0.44	0.00	0.03	0.41	0.89
Physicians should try to understand what is going on in their patients’ minds by paying attention to their non-verbal cues and body language.(13)	0.40	0.17	0.04	0.49	1.2
Empathy is a therapeutic skill without which the physician’s success is limited.(15)	0.36	0.20	-0.04	0.44	1.2
Understanding body language is as important as verbal communication in physician-patient relationships.(4)	0.30	0.09	0.08	0.35	0.88
A physician’s sense of humor contributes to a better clinical outcome.(5)	0.29	0.03	0.00	0.26	0.79
Patients’ illnesses can be cured only by medical or surgical treatment; therefore, physicians’ emotional ties with their patients do not have a significant influence in medical or surgical treatment.(11)	0.03	0.59	0.01	0.52	1.2
I believe that emotion has no place in the treatment of medical illness.(14)	0.23	0.54	0.04	0.46	1.0
Attentiveness to patients’ personal experiences does not influence treatment outcomes.(8)	0.01	0.52	0.05	0.48	1.1
Asking patients about what is happening in their personal lives is not helpful in understanding their physical complaints.(12)	0.03	0.49	0.00	0.44	1.0
Physicians’ understanding of their patients’ feelings and the feelings of their patients’ families does not influence medical or surgical treatment.(1)	0.04	0.49	-0.09	0.35	0.94
Attention to patients’ emotions is not important in history taking.(7)	0.01	0.48	0.09	0.43	1.0
I do not enjoy reading non-medical literature or the arts.(19)	0.00	0.25	0.00	0.20	0.62
Physicians should not allow themselves to be influenced by strong personal bonds between their patients and their family members.(18)	-0.02	0.21	0.01	0.13	0.50
Because people are different, it is difficult to see things from patients’ perspectives.(6)	-0.05	0.06	0.75	0.15	0.59
It is difficult for a physician to view things from patients’ perspectives.(3)	0.06	-0.06	0.68	0.14	0.57

*Principal component factor extraction with oblique rotation was used for approximately half of the sample (n=1380). Confirmatory factor analysis was performed for the other half of the sample to examine the 3-factor model.

†Items are listed by the order of magnitude of factor loadings within each extracted factor. Factor loadings equal to or greater than 0.25 are in bold. Numbers in parentheses represent the sequence of the items in the actual scale. Items were scored using a 7-point Likert-type scale. Half of the items are reverse scored.

‡These are partial correlations between score of each item and total JSE score by excluding the corresponding item score from the total score. Item-total score correlations and discrimination indices were calculated based on data for the entire sample (N=2612). For calculation of the effect size estimates of discrimination indices, the item mean score for JSE high scorers (top 33%) was subtracted from the item mean score for JSE low scorers (bottom 33%), divided by the pooled standard deviation of the corresponding item.

**p <0.001 for all of the reported correlations.

### Item discrimination indices

The item discrimination effect size indices ranged from a low of 0.50 for the aforementioned item which showed the lowest item-total score correlation, to a high of 1.4 for the above-mentioned item with the highest item-total score correlation. The median effect size was 1.2 (See Table 1). Cohen^46^ suggests that the effect size values around 0.30 or lower are considered negligible, around 0.50 are moderate, and around 0.70 and higher are large and practically important. According to these operational definitions, the item discrimination effect size indices were all substantial, and practically important.^48^

### Internal consistency aspect of reliability

The internal consistency aspect of reliability of the entire JSE was determined by Cronbach’s coefficient α, which was 0.80, (95% CI [0.79-0.84]), ranging from a low of 0.75 (for matriculants of 2006) to a high of 0.84 (for matriculants of 2008 and 2009). Reliability coefficients of these magnitudes are considered acceptable by professional organizations such as the American Educational Research Association, American Psychological Association, and National Council on Measurement in Education.^49^ Reliability coefficients in 0.70s and 0.80s magnitudes have often been reported in almost all of the JSE studies in the US and abroad.

### Reaffirming the underlying components of the JSE

We re-examined the underlying components of the JSE by using exploratory factor analysis. In almost all of the factor analytic studies cited previously orthogonal (varimax) rotation was used to obtain independent factors. In the present study, we used oblique rotation (promax) to allow correlations among the extracted factors in order to examine if previously reported factor patterns would remain unchanged. We also limited the number of retained factors to three to make the findings comparable to the previously reported factor analytic results.^9^^,^^17^ Indeed, scree test to determine the appropriate number of factors to retain before rotation showed that the plot of the eigenvalues leveled off after extraction of the third factor, supporting our decision to retain three factors for rotation. The Kaiser-Meyer-Olkin measure for sampling adequacy (MSA) was used prior to factor extraction which resulted in an overall index of 0.86, supporting the adequacy of data for factor analysis. Also, the Bartlett’s test for sphericity showed that the intercorrelation matrix was factorable (χ^2^ (190) = 5332.5, p <0.0001).

The eigenvalues for the first, second, and third retained factors were 4.7, 1.6, and 1.4, respectively. The first factor, “perspective-taking,” included 10 items with factor coefficients greater than 0.25, accounting for 23% of the total variance. A sample item (with the highest factor coefficient) is: “Patients value a physician’s understanding of their feelings which is therapeutic in its own right.” The Cronbach’s coefficient alpha reliability for items under this factor was 0.79 (95% CI [0.78-0.81]). The second factor, “compassionate care,” included seven items with factor coefficients of 0.25 or greater, accounting for 8% of the total variance. A sample item is: “Patients’ illnesses can be cured only by medical and surgical treatment; therefore, physicians’ emotional ties with their patients do not have influence in medical or surgical treatment.” This is a negatively worded item which is reverse scored. The Cronbach’s coefficient alpha reliability for items under this factor was 0.69 (95% CI [0.67-0.71]). The third factor includes only two items with factor coefficients greater than 0.67, accounting for 7% of the total variance. A sample item is: “Because people are different, it is difficult to see things from patients’ perspectives” (reverse scored). The Cronbach’s coefficient alpha reliability for items under this factor was 0.68 (95% CI [0.65-0.70]). One item had a low factor coefficient (0.21) on Factor 2. However, this item showed a significant item discrimination effect size index and yielded a statistically significant (but low in magnitude) item-total score correlation. Summary results of the EFA are reported in Table 1.

The general pattern of EFA findings is similar to most other studies in the US and abroad. For example, similarities in factor pattern are observed in studies reported for the physicians;^17^ and nurses^27^ in the Unites States, and for samples of physicians in Italy; ^50^ medical students in Iran; ^45^ Korea;^32^ Japan;^31^ Mexico;^30^ South Africa;^33^ mainland China;^35^ Taiwan;^36^ Brazil;^40^ Austria;^44^ and England.^41^ The two factors of “perspective-taking” and “compassionate care” emerged in almost all of the factor analytic studies of the JSE. Similarities in factor pattern of the JSE in different samples and in different countries indicate that the underlying components of the scale are relatively stable, regardless of cultural variation.

### Confirming the latent variable structure of the JSE

**Figure 1:**
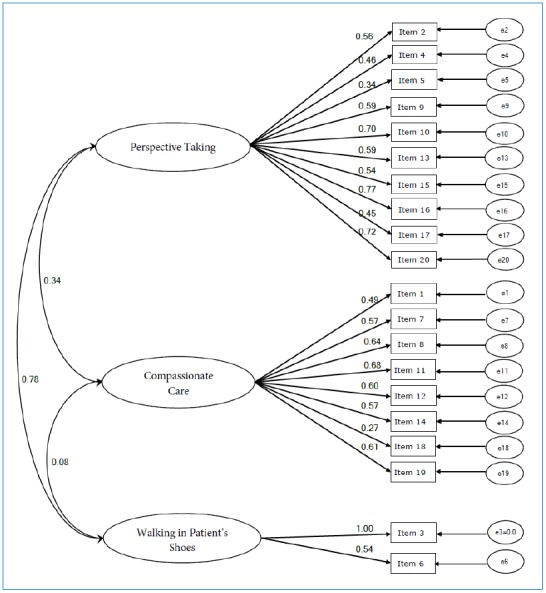
Three-Factor Model (Latent Variable Structure) of the Jefferson Scale of Empathy (n=1,232)

In confirmatory factor analysis, all 20 items were modeled as functions of three underlying latent variables which emerged in the exploratory factor analysis and have been widely reported. Maximum likelihood (ML) estimation was used. The regression coefficient for one item-to-latent variable path for each latent variable was set to 1.0 to scale the latent variable. Additionally the variance of one error term (that corresponding to item 6) was set to 0.0 to facilitate convergence of the ML estimation. Without this constraint, the model was inadmissible due to the negative error variance of this item.^51^ Also, owing to previous studies cited above, as well as the validation study of the JSE, the covariances of all latent variables were also estimated. The model was identified with 134 degrees of freedom.As an exploratory analysis, we also evaluated a 2-factor model; one which omitted the two items which comprise factor 3–“walking in patient’s shoes.” This was done because of the failure of the maximum likelihood CFA to converge without constraining one error variance, which can indicate a mis-specified model,^51^ and the other CFA studies of the scale which modeled only 2 factors.^41^^,^^43^ We compared the fit of this two-factor model to the fit of the three-factor model.Assessment of model fit was made through the use of several well-accepted metrics in structural equation modeling (SEM). First, the χ^2^ test for the model was reviewed. In SEM, it is a measure of fit, rather than a test statistic, and desired values are small and non-significant. However, since χ^2^ is sensitive to sample size, it is possible to obtain a large and significant value even when the fit of the model to the data is acceptable. To address this, a widely used ‘rule of thumb’ was also evaluated – the ratio of the χ^2^ to its degrees of freedom, which is suggested to reflect good fit at values < 4.0.^52^ We also evaluated the adjusted ‘goodness of fit’ index (AGFI) which indexes the proportion of the observed covariance matrix that is explained by the model-implied covariance matrix.^53^ The Tucker-Lewis Index (TLI) was used to compare the fitted model to a null model. Hu and Bentler^54^ recommend values >0.95. Finally, the RMSEA (root mean square error for approximation) for the structural model was evaluated. Hu and Bentler^54^ showed that a cutoff of 0.06 for RMSEA indicates good model fit.For model comparisons, an additional fit and an incremental fit improvement metrics were used. The models were first compared to each other through the use of a χ^2^ test for the significance of the difference in fit. The non-normed fit index (NNFI; also known as the TLI: Tucker-Lewis Index) was used to assess improvements in fit from model to model. The TLI normally results from SEM output as a comparison to a “null” model, but a version can be calculated for the improvement in fit between any two competing models. Hu and Bentler^54^ suggested that improvements in the TLI greater than 0.02 are of “substantive interest”. See Figure 1 for the measurement model structure of 20 variables and three correlated factors.

The 2-factor solution did not show a good fit (RMSEA=0.07, AGFI=0.88); however, the 3-factor confirmatory factor analysis yielded a marginally good fit to the data; RMSA=0.05 and AGFI greater than 0.90. Both the χ^2^ difference test, and the TLI suggest that the 3 factor model is a better fit than the two factor model.

Of note in this analysis is that the error variance for item 6 (one indicator of the “walking in the patient’s shoes” latent variable) had to be constrained in order to reach model converge. Overall model fit is acceptable for the 3-factor model, as reflected specifically by the RMSEA value, which has been suggested to be more important reflection of model specification for a purely structural model like this one.^55^ In addition, evaluation of a model which completely omitted this latent variable showed a poor fit to the data. Coupled with the fact that other CFA models of the scale have found a 3-factor structure, we suggest that there is a reliable 3^rd^ latent variable underlying these items. Because the initial process of item generation for the scale was not predicated on the idea that there would be 3 latent variables, it is possible that enough items were not generated for the third factor which is a reliable component of self-report empathy in the context of patient care. We therefore suggest retaining all of the 20 items in the instrument not only for the goodness of the fit of the 3-factor model, but also because of significant item-total score correlations and substantial item discrimination effect size indices obtained for all items. Table 2 shows summary results for fit statistics.

**Table 2:** Summary results of confirmatory factor analysis fit statistics (n=1,232)

Model	Fitted 3-factor model	Fitted 2-factor model	Difference	Null model (1 factor model)
Parameter estimate	42	36		20
χ^2^	887.87	984.51	205.65	6469.32
df	168	135	33*	190
χ^2^/*df*	5.28	7.29		34.05
AGFI	0.93	0.88		0.39
TLI	0.89	0.843	0.4^a^	0
RMSEA	0.05	0.071		0.16
AIC	971.87			7468.25

*p<0.05.

^a^Calculated as recommended in Hu & Bentler,^54^ this value represents a significant improvement in fit over the two-factor model.

Results of CFA support the 3-factor model of the JSE, and are in agreement with those reported in Iranian medical students,^45^ and British medical students.^41^ A satisfactory 3-factor model fit was also achieved in Portuguese medical students after relaxing model restrictions.^37^ The 2-factor model (“perspective-taking” and “compassionate care”) in Australian paramedic students^43^ partly resembles findings of the present study. Although we acknowledge that these findings overall (including the current study) are not definitive with regard to the structure of the scale, we do not agree with suggestions made by some that a few JSE items should be excluded for a better latent variable structure model.^43^ First, deletion of items can cause an incompatibility problem in comparative research. Second, in most of the psychometric studies of the JSE (including the present study), significant item-total score correlations have been reported suggesting that each item contributes significantly to the total score of the JSE. In addition, we showed in this study that each item can discriminate substantially between high and low scorers of the JSE.

## Discussion

The JSE was developed in response to a need for a psychometrically sound instrument to measure empathy in the context of medical education and patient care. Although extensive support for its validity and reliability is available, further evidence in support of its underlying components and its latent variable structure provides additional support for the construct validity of the scale.Examination of data in this large scale study supported the previously reported findings on the reliability (Cronbach’s α), underlying constructs, and confirmation of the latent variable structure of the JSE. Similarities in factor pattern of the JSE in different samples and in different countries indicate that the underlying components of the scale are relatively stable, regardless of cultural variation. The three components of “perspective-taking”, “compassionate care”, and “walking in patient’s shoes” which emerge in this and some other factor analytic studies of the JSE are consistent with the ingredients of empathy often reported in the literature.^9^ These underlying factors are also supportive of the pillars of empathic engagement in patient care described elsewhere;^9^ namely, seeing with the mind’s eye (e.g., perspective-taking, and walking in patient’s shoes) and hearing with the third ear (e.g., compassionate care). Based on the findings from the CFA, we suggest to retain all of the 20 items in the instrument not only for the goodness of the fit of the 3-factor model, but also because of significant item-total score correlations and substantial item discrimination effect size indices obtained for all items.Needless to say that psychometric properties of an attribute, such as empathy in patient care, can be a function of several factors including sociocultural, educational, and environmental factors^2^ which necessitate a continued effort to examine psychometrics of the JSE in different sociocultural environment, populations, and in different translated versions of the scale to assure the psychometric soundness of the JSE in a variety of situations. Such broad psychometric support would add to the credibility of the JSE and raise confidence of its users wherever it is applied.

### Limitations

As noted above, this study did not conclusively support a 3-factor latent variable scale structure for the JSE. Further exploratory studies may be desirable to further explore this issue in different samples of health profession students and practitioners. In this sample, we noticed a ceiling effect, or relatively high mean scores (>6.0) across 7 items, which may have contributed to the marginal model fit.

## 

### Conflict of Interest

The authors declare that they have no conflict of interest.
